# In Vitro Anti-HIV-1 Reverse Transcriptase and Integrase Properties of *Punica granatum* L. Leaves, Bark, and Peel Extracts and Their Main Compounds

**DOI:** 10.3390/plants10102124

**Published:** 2021-10-07

**Authors:** Cinzia Sanna, Arianna Marengo, Stefano Acquadro, Alessia Caredda, Roberta Lai, Angela Corona, Enzo Tramontano, Patrizia Rubiolo, Francesca Esposito

**Affiliations:** 1 Laboratory of Pharmaceutical Botany, Department of Life and Environmental Sciences, University of Cagliari, Via S. Ignazio da Laconi 13, 09123 Cagliari, Italy; roberta.lai@unica.it; 2 Department of Drug Science and Technology, University of Turin, Via Pietro Giuria 9, 10125 Turin, Italy; arianna.marengo@unito.it (A.M.); stefano.acquadro@alice.it (S.A.); patrizia.rubiolo@unito.it (P.R.); 3 Laboratory of Molecular Virology, Department of Life and Environmental Sciences, University of Cagliari, Cittadella Universitaria di Monserrato, ss554, km 4500, Monserrato, 09042 Cagliari, Italy; alessiacaredda@unica.it (A.C.); angela.corona@unica.it (A.C.); tramon@unica.it (E.T.); francescaesposito@unica.it (F.E.)

**Keywords:** *Punica granatum*, pomegranate, leaves, bark, peel, HIV-1 reverse transcriptase, HIV-1 integrase, ellagic acid, hydrolyzable tannins, triterpenoids

## Abstract

In a search for natural compounds with anti-HIV-1 activity, we studied the effect of the ethanolic extract obtained from leaves, bark, and peels of *Punica granatum* L. for the inhibition of the HIV-1 reverse transcriptase (RT)-associated ribonuclease H (RNase H) and integrase (IN) LEDGF-dependent activities. The chemical analyses led to the detection of compounds belonging mainly to the phenolic and flavonoid chemical classes. Ellagic acid, flavones, and triterpenoid molecules were identified in leaves. The bark and peels were characterized by the presence of hydrolyzable tannins, such as punicalins and punicalagins, together with ellagic acid. Among the isolated compounds, the hydrolyzable tannins and ellagic acid showed a very high inhibition (IC_50_ values ranging from 0.12 to 1.4 µM and 0.065 to 0.09 µM of the RNase H and IN activities, respectively). Of the flavonoids, luteolin and apigenin were found to be able to inhibit RNase H and IN functions (IC_50_ values in the 3.7–22 μM range), whereas luteolin 7-*O*-glucoside showed selective activity for HIV-1 IN. In contrast, betulinic acid, ursolic acid, and oleanolic acid were selective for the HIV-1 RNase H activity. Our results strongly support the potential of non-edible *P. granatum* organs as a valuable source of anti-HIV-1 compounds.

## 1. Introduction

*Punica granatum* L. (Pomegranate) is a shrub or small tree belonging to the Lythraceae family that originated from Central Asia, from where it spread to the rest of the world [1]. Since ancient times, it has been cultivated in the Mediterranean region. Today, over 500 varieties are recognized [2]. Pomegranate fruits, pericarp, flowers, and root bark have been used to treat a variety of ailments in Chinese, Ayurvedic, Islamic, and Persian medicine [1,3,4]. More recently, it has been considered a functional food due to a variety of active components, including tannins, alkaloids, organic acids, flavonoids, and terpenes [2], endowed with a wide range of pharmacological effects. Interestingly, the nutraceutical value is not limited to the fruits; in fact, the non-edible fractions of the fruits and tree (peels, seeds, bark, and leaves), which are discarded or disregarded, might be a valuable source of phytochemicals [5,6], promoting the exploitation and valorization of agricultural wastes and byproducts enriched in high added-value molecules [7]. Fruits (peel and juice) are rich in phenolic compounds, including flavonoids and hydrolyzable tannins (punicalin, pedunculagin, punicalagin, gallic acid, and ellagic acid), and flavonols (quercetin and myricetin) [1,8]. Moreover, anthocyanins, such as pelargonidin, cyanidin, and delphinidin glucosides, have been reported in both edible and inedible parts of fruits [9,10]. In addition to the hydrolyzable tannins identified in fruit peel, bark contains C-glycosidic ellagitannins, such as punicacorteins A–D, punigluconin, casuariin, and casuariniin [11], and dimeric gallic acid glycosides [12]. Leaves contain many gallic acid derivatives, also known as gallotannins and ellagitannins [3,13], and phenolics and flavone glycosides [3,14]. Both qualitative and quantitative differences have been observed in different pomegranate cultivars [9,15] as well as seasonal changes in their content [16]. Due to its bioactive compounds, pomegranate possesses antioxidant, anti-inflammatory, antidiabetic, anticancer, antimicrobial, cardioprotective, and neuroprotective effects [1,5,6,17,18,19,20,21]. Antiviral properties against human herpes virus 3 (HHV-3) [22], herpes simplex virus 2 (HSV-2) [23], influenza virus [24], and enterovirus 71 [25] have been reported and, recently, Acquadro et al. 2020 [14] tested the extract of pomegranate leaves from Sardinia for its antiviral properties against Zika virus. Moreover, pomegranate fruit juice contains molecules that target the virus envelope and are able to prevent HIV-1 binding to the cellular receptor CD4 and CXCR4/CCR5 [26]. Despite the availability of many target-specific anti-HIV-1 drugs, their efficacy is often compromised by their toxicity and the selection of drug-resistant viral strains. Therefore, the discovery of new compounds with a novel mechanism of action is needed. In this context, the development of multi-target inhibitors would provide a relevant aid [27,28].

With the aim of finding plant compounds that function as dual HIV-1 inhibitors, in this study we investigated the ability of ethanol extracts and pure compounds obtained from leaves, bark, and peel of *P. granatum* to inhibit in enzymatic assays the HIV-1 reverse transcriptase (RT)-associated ribonuclease H (RNase H) activity, an attractive target for which there is currently no approved drug [29,30,31]. Therefore, considering its similarity to RNase H [31], the inhibition of integrase (IN) in the presence of the LEDGFp75 cellular cofactor was also evaluated. Due to its pivotal role in virus replication, the interaction between LEDGF/p75 and IN is an attractive target for a novel anti-retroviral strategy. In fact, LEDGFp75 tethers IN associated with the viral genome to the host chromatin, promoting the integration process [32,33]. Interestingly, extensive depletion of LEDGF/p75 levels was correlated to a significant decrease in HIV-1 infectivity.

## 2. Results and Discussion

Fruits, peel, and bark of *P. granatum* have been used in traditional medicine to treat a variety of ailments. The great interest in the medicinal and nutritional value of pomegranate is documented by the >1500 articles on its health effects that have been published to date. However, despite the relatively large amount of literature on pomegranate fruits (juice and peel), much less information is available regarding the other organs. In our search for anti-HIV-1 agents from plants [28,34,35,36,37,38,39], we firstly tested the ethanolic extracts obtained from the leaves (PGL), bark (PGB), and peels (PGP) of *P. granatum* from Sardinia for their ability to inhibit the RT-associated RNase H activity. This is regarded as an attractive and innovative target, since even though the majority of anti-AIDS molecules available for therapy are represented by RT inhibitors, all of them inhibit its associated DNA polymerase activity. Subsequently, considering the high degree of structural homology between the RNase H catalytic core and HIV-1 IN [29], the inhibition of the HIV-1 IN strand transfer reaction in the presence of LEDGF/p75 was also evaluated. In fact, compounds inhibiting the HIV-1 RNase H activity may also influence the HIV-1 IN activity [30]. The development of dual inhibitors, i.e., single molecules targeting more than one enzyme function, has recently been proposed as an original approach, since it potentially reduces the side effects associated with the coadministration of different classes of drugs [40,41].

### 2.1. Anti-HIV-1 Activity of Ethanolic Extracts Obtained from P. granatum Bark (PGB), Leaves (PGL), and Fruit Peels (PGP)

All the pomegranate extracts showed a strong inhibitory activity on both enzymes, with IC_50_ values ranging from 0.22 to 0.85 µg/mL on HIV-1 RT RNase H activity and from 0.12 to 0.5 µg/mL on HIV-1 IN LEDGF-dependent activity, respectively (Table 1).

Among the three extracts, PGB exerted the highest inhibitory activity against HIV-1 RT RNase H, followed by PGL and PGP. On the other hand, PGL was more active against HIV-1 IN LEDGF-dependent activity, followed by PGB and PGP.

### 2.2. Phytochemical Study of Punica granatum Extracts

**Figure 1:**
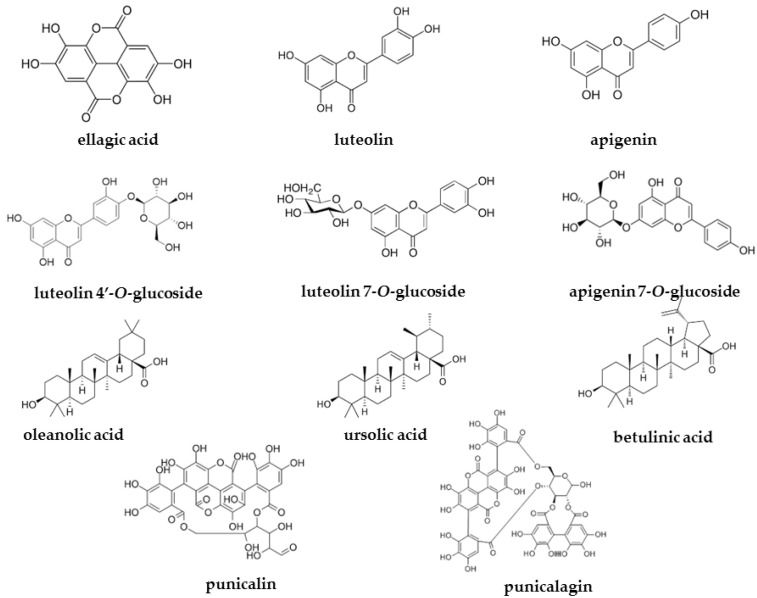
Compounds detected in leaves, bark, and fruit peel ethanolic extracts of *P. granatum* L.

In order to identify the main compounds in PGL, PGB, and PGP extracts, a phytochemical study was undertaken. HPLC-PDA-MS/MS analyses evidenced compounds belonging to the phenolic and flavonoid chemical classes (Figure 1).

A list of the identified and putatively identified compounds in the PGB and PGP extracts is shown in Table 2. The representative compounds in the PGL extract were reported in Acquadro et al. 2020 [14].

According to the literature, ellagic acid is the most abundant specialized metabolite in pomegranate leaves, together with flavones, particularly luteolin and apigenin glycosides [20,48]. Moreover, the presence of triterpenoid molecules was confirmed by GC-MS analyses of the derivatized extract, which led to the identification of oleanolic acid, betulinic acid, and ursolic acid.

**Figure 2:**
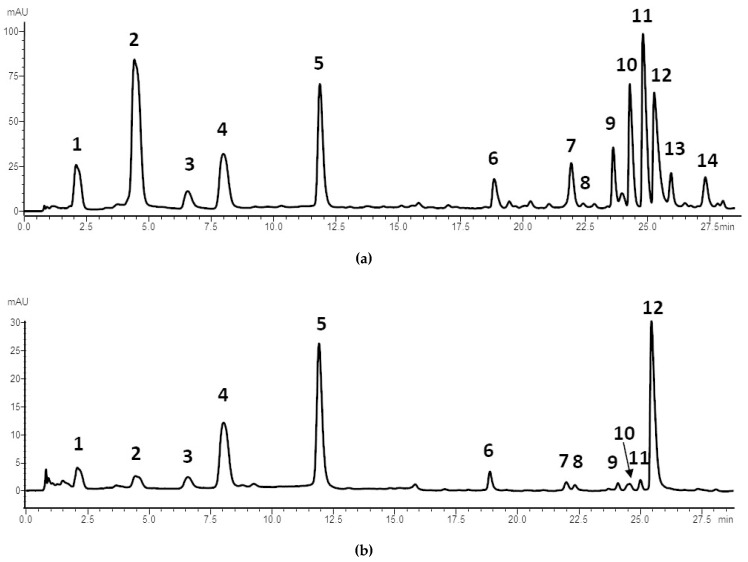
Chromatographic profiles of PGB (**a**) and PGP (**b**) (λ = 370 nm). The compounds are numbered according to Table 2.

Bark and peels were characterized, in accordance with the available data [1,11], by the presence of hydrolyzable tannins together with ellagic acid (Figure 2).

In particular, punicalagins and punicalins, which were not detected in leaves, are considered to be markers of these plant organs. Punicalagin isomers are known to constitute up to 85% of the total tannins extracted from pomegranate fruit peels [15].

A comparison of the PGF, PGB, and PGP LC-PDA profiles is reported in Figure S1.

### 2.3. Anti-HIV-1 Activity of Pure Compounds Detected in PGB, PGL, and PGP

To verify the contribution of individual identified components of the pomegranate leaves, bark, and peel extracts to the inhibition of HIV-1 RT-associated RNase H and IN LEDGF-dependent activities, their effect on these enzymes was evaluated and their IC_50_ values are shown in Table 3.

Consistent with our findings, effects of ellagic acid on HIV-1 IN activity have previously been observed [49,50]. Ellagic acid was also found to be able to suppress the replication of X4-tropic HIV-1 in the target cells without cytotoxicity [50]. This compound was also reported to have the ability to inhibit HIV-1 protease expression [51]. Differently from our results, Nutan et al. 2013 [51] reported the inability of ellagic acid to inhibit HIV RT. However, they assayed the ability of ellagic acid to inhibit RT DNA polymerase activity, not RNase H activity. This is a possible reason for the discrepancy with respect to our data on HIV RT inhibitory activity.

Luteolin and apigenin, two flavones detected in the PGL extract, were found to be active on both HIV-1 enzymes, showing IC_50_ values of 3.7 µM and 16.1 µM for RNase H activity and 6.5 µM and 22 µM for HIV-1 IN LEDGF-dependent activity, respectively, in agreement with our previous studies [38] although we found a lower degree of inhibition compared with ellagic acid and the control. Flavonoids are a class of natural compounds endowed with a broad spectrum of biological activities, including anti-SARS-CoV-2 activity [52] and promising anti-HIV-1 properties [53,54]. Interestingly, luteolin was found to be capable of inhibiting HIV-1 infection in Jurkat cells, showing better inhibition when added after HIV-1 infection, indicating that it exerts antiviral activity in the later stages of the HIV-1 lifecycle [55].

Recently, some flavonoid-based HIV-1 IN inhibitors targeting both the active site and the interaction with LEDGF/p75 were designed by introducing hydrophobic moieties into the flavanol core [56]. Out of the three luteolin and apigenin glycosides identified in PGL, only luteolin 7-*O*-glucoside was able to inhibit the HIV-1 IN LEDGF-dependent activity, with an IC_50_ value of 8.5 µM, showing a selective profile for this enzyme, while luteolin 4′-*O*-glucoside and apigenin 7-*O*-glucoside were found to be totally inactive at the highest tested concentration (Table 3). The three triterpenes, oleanolic acid, ursolic acid, and betulinic acid, identified in PGL were able to inhibit RT-associated RNase H activity, with IC_50_ values in the 2–7 μM range, whereas they were found to be inactive, or weakly active in the case of betulinic acid, on the HIV-1 IN LEDGF-dependent activity. Betulinic acid has previously been reported to inhibit different steps of the HIV-1 lifecycle [57,58]. In particular, it inhibited HIV-1 RT-associated functions of both wild-type and mutant RTs [35]. Since their structures are partially correlated, betulinic acid, oleanolic acid, ursolic acid, and some of their derivatives were identified as anti-HIV principles, even though ursolic acid is slightly toxic [59]. Moreover, ursolic acid and oleanolic acid have been reported to inhibit protease expression [60,61].

Punicalins and punicalagins, which were detected in both PGB and PGP extracts, were found to be able to inhibit both enzymes and, in particular, the HIV-1 IN LEDGF-dependent activity, with IC_50_ values comparable to those exhibited by Raltegravir, which was used as a positive control. These compounds inhibited HIV-1 replication in infected H9 lymphocytes with a low degree of cytotoxicity [62]. In particular, punicalins have been found to be able to inhibit RT-associated RNase H activity [63] and to block the binding of HIV rgpl20 to CD4 [64].

Our findings suggest the possible utilization of non-edible organs of *P. granatum* as a source of valuable therapeutic agents. Interestingly, of the specialized metabolites identified, ellagic acid, luteolin, apigenin, punicalins, and punicalagins, whose quantitative data in PGL, PGB, and PGP extracts are reported in Table 4, could be used in the development of new multi-target drugs, since they are capable of inhibiting both HIV-1 enzymes. In fact, they represent a starting point for the development of new chemical derivatives whose modification could lead to new compounds with a multi-target function.

## 3. Materials and Methods

### 3.1. Plant Material

Fresh leaves, bark, and fruit peel of *P. granatum* were collected from the botanical garden of the University of Cagliari (Sardinia, Italy). Samples were authenticated by Dr. Cinzia Sanna from the Department of Life and Environmental Sciences, where a voucher specimen (*Herbarium* CAG 514/A) was deposited. Plant material was dried in a ventilated stove at 40 °C to constant weight.

### 3.2. Chemicals

HPLC-grade methanol, LC-MS-grade acetonitrile, formic acid (>98% purity), pyridine, BSTFA, ellagic acid, rutin, and apigenin were obtained from Merck (Darmstadt, Germany). De-ionized water (18.2 MΩ cm) was supplied by a Milli-Q purification system (Millipore). Luteolin, luteolin 7-*O*-glucoside, luteolin 4′-*O*-glucoside, apigenin 7-*O*-glucoside, quercetin 3-*O*-glucoside, betulinic acid, oleanolic acid, and ursolic acid were purchased from Extrasynthese (Genay, France). Punicalin α+β and punicalagin α+β were obtained from Phytolab (Vestenbergsgreuth, Germany).

The reagents used for expression, purification, and biochemical assays were purchased from Microbiol (Sardinia, Italy), Sigma-Aldrich (Milano, Italy), and PerkinElmer (Milano, Italy). The reference compound Raltegravir was purchased from ChemScene (Monmouth Junction, United States), while the reference compound RDS1759 was kindly provided by Prof. Roberto Di Santo (University of Rome La Sapienza). Recombinant proteins were purified using a Biological LP chromatography system (Biorad).

### 3.3. Extraction and Detection of Active Compounds

Dried leaves (115 g), bark (150 g), and peel (100 g) of *P. granatum* were separately ground in an electric grinder and the obtained powder was extracted with 1 L of EtOH 100% for 8 h. The procedure was repeated three times. The extracts were then combined, filtered, and evaporated to obtain crude extracts (13.9 g, 3.5 g, and 10.4 g, respectively). The characterization of the pomegranate extracts was carried out by HPLC-PDA-MS/MS using a Shimadzu Nexera X2 system coupled to an SPD-M20A photodiode array detector connected in series to a Shimadzu LCMS-8040 triple quadrupole system equipped with an ESI source (Shimadzu) under the operation conditions described in [14]. The main components were identified by comparison of MS fragmentation data with those reported in the literature and, when available, with the co-injection of the relative commercial standards. The quantitative data were obtained through an external calibration curve acquired via UV (at the λ max for each compound) and single reaction monitoring (SRM) acquisition in ESI^+^ (collision energy, 35.0 V; dwell time, 20). The quantitative parameters are reported in Table S1.

GC-MS analyses were carried out on pomegranate leaf extracts after derivatization with bis(trimethylsilyl)trifluoroacetamide to obtain trimethylsilyl derivatives, as reported by Rubiolo et al. 2013 [64]. The GC-MS characteristics and operation conditions have previously been described in Acquadro et al. 2020 [14].

### 3.4. Expression and Purification of Recombinant HIV-1 RT

HIV-1 group M subtype B heterodimeric RT was expressed and purified essentially as previously described [65,66].

### 3.5. Expression and Purification of Recombinant HIV-1 IN and LEDGF/p75 Proteins

Recombinant IN and LEDGF/p75 proteins were expressed and purified as described by Esposito et al. 2020 [67].

### 3.6. RNase H Polymerase Independent Cleavage Assay

HIV RT-associated RNase H activity was measured as described by Corona et al. 2016 [68] using the RNase H inhibitor RDS1759 as a control. Data analysis of the assay results was performed using GraphPad Prism 9.

### 3.7. Homogeneous Time-Resolved Fluorescence (HTRF) IN LEDGF-Dependent Assay

The IN activity was measured in the presence of LEDGF/p75 cellular cofactor in an HTRF assay [69,70]. Data analysis of the assay results was performed using GraphPad Prism 9.

## 4. Conclusions

This study reports the significant anti-HIV-1 activity of crude extracts and pure compounds detected in *Punica granatum* leaves, bark, and peel. Phytochemical investigation led to identification of the main compounds, which are, according to the available literature, hydrolyzable tannins, phenolics and flavone glycosides, and triterpenoids. Of the identified specialized metabolites, ellagic acid, punicalins, and punicalagins exhibited the most relevant inhibition of RT-associated HIV-1 RNase H and HIV-1 IN LEDGF-dependent activities. Consistent with the literature data, the betulinic acid, oleanolic acid, and ursolic acid identified in the pomegranate leaves were also found to be active, although only on the HIV-1 RT-associated RNase H function. This lead, therefore, to a special interest in ellagic acid and ellagitannins since their dual activity, on both enzymes, could be exploited in the development of derivatives with multi-target functions to avoid the use of several drugs with different activities. Our findings, therefore, suggest a possible use for and valorization of non-edible parts of *P. granatum* in line with the circular economy concept. Leaves, bark, and peels are in fact often considered to be agricultural wastes or byproducts; however, their enrichment in phytochemicals could be used in the development of drugs to treat HIV infection.

## Figures and Tables

**Table 1:** Effect of *P. granatum* extracts on the HIV-1 RT-associated RNase H function and Integrase activity in the presence of LEDGF.

Extracts/Pure Compounds	HIV-1 RT-Associated RNase H IC_50_ (µg/mL) ^a^	HIV-1 Integrase LEDGF-Dependent IC_50_ (µg/mL) ^b^
*Punica granatum* leaves extract (PGL)	0.61 ± 0.02	0.12 ± 0.065
*Punica granatum* bark extract (PGB)	0.22 ± 0.04	0.18 ± 0.02
*Punica granatum* peel extract (PGP)	0.85 ± 0,01	0.5 ± 0.035
RDS1759	10.7 ± 0.9^c^	-
Raltegravir	-	0.058 ± 0.01 ^c^

^a^ Extract concentration required to reduce the HIV-1 RT-associated RNase H activity by 50%. ^b^ Extract concentration required to inhibit the HIV-1 IN catalytic activity by 50% in the presence of LEDGF. ^c^ Compound concentration expressed in µM.

**Table 2:** Identified and hypothesized compounds in PGB and PGP extracts. For each compound, the relative retention time (RT), maximum absorption (nm), molecular formula, pseudomolecular ions (ESI^+^ and ESI^−^), fragments obtained in product ion scan mode (PIS), and identified or putatively identified compound names are indicated. The identification confidence value and the bibliography reference related to the detected compounds in pomegranate are also reported.

N°	RT	λmax (nm)	Molecular Formula	[M+H]^+^	[M−H]^−^	Supposed MW	MS^2+^ *m/z*	MS^2−^ *m/z*	Compound Name	Identif. Confidence ^a^	Ref.
1	2.076	257/377	C_34_H_22_O_22_	783	781	782	603	601	Punicalin α + β	1	[42]
2	4.414	258/376	C_48_H_28_O_30_	1085	1083	1084	603	601	Punicalagin isomer	2	[43]
3	6.553	257/370	C_21_H_10_O_13_	471	469	470	453, 407, 151, 363	451, 425, 353, 341	Valoneic acid dilactone	2	[44]
4	7.984	257/376	C_48_H_28_O_30_	1085	1083	1084	621, 603	601	Punicalagin α	1	[42]
5	11.853	257/380	C_48_H_28_O_30_	1085	1083	1084	765, 621, 603	781, 721, 301	Punicalagin β	1	[42]
6	18.842	252/360	C_20_H_16_O_13_	465	463	464	345, 315, 303, 285, 223	301, 283	Ellagic acid glucoside	2	[45]
7	21.931	254/364	C_21_H_10_O_13_	471	469	470	407, 303, 168, 139	301, 271, 227, 201, 171	Sanguisorbic acid dilactone	2	[46]
8	22.859	252/364		435	433	434	303, 285, 275	301	Ellagic acid derivative	3	
9	23.613	251/360	C_19_H_14_O_12_	435	433	434	303, 285	301, 283	Ellagic acid-pentoside	2	[42]
10	24.279	251/360	C_20_H_16_O_12_	449	447	448	303, 285, 273	301, 257, 229	Ellagic acid deoxyhexoside	2	[45]
11	25.409	255/373		1067	1065	1066	603, 575	/	Ellagitannin	3	
12	25.658	252/366	C_14_H_6_O_8_	303	301	302	/	284, 229, 185	Ellagic acid	1	[42]
13	25.934	255/372			301			275, 256, 127	Ellagitannin	3	
14	27.309	256/379	C_28_H_10_O_16_		601			101	Gallagic acid dilactone	2	[46]

^a^ Identification confidence is based on CAWG (2007) [47]: Level 1: Identified compound (at least two independent orthogonal data values, such as retention time and mass spectrum, in accordance with an authentic reference standard); Level 2: Tentatively characterized compound (a compound identified by a comparison of spectral data and bibliographic data); Level 3: Putatively annotated class compound; Level 4: unknown compound.

**Table 3:** Effect of *P. granatum* extracts on the HIV-1 RT-associated RNase H function and Integrase activity in the presence of LEDGF.

Pure Compounds	HIV-1 RT-Associated RNase HIC_50_ (µM) ^a^	HIV-1 Integrase LEDGF-Dependent IC_50_ (µM) ^b^
Ellagic acid	1.4 ± 0.11	0.075 ± 0.0005
Luteolin	3.7 ± 0.5	6.5 ± 0.5
Apigenin	16.1 ± 0.6	22 ± 3.5
Luteolin 4′-*O*-glucoside	>100 (100%) ^c^	>100 (100%) ^c^
Luteolin 7-*O*-glucoside	>100 (100%) ^c^	8.5 ± 1.4
Apigenin 7-*O*-glucoside	>100 (100%) ^c^	>100 (100%) ^c^
Oleanolic acid	6.7 ± 0.4	>100 (74%) ^c^
Ursolic acid	5.7 ± 0.1	>100 (68%) ^c^
Betulinic acid	2.0 ± 0.2	96.5 ± 3.5
Punicalins	0.18 ± 0.03	0.09 ± 0.01
Punicalagins	0.12 ± 0.00	0.065 ± 0.00

^a^ Compound concentration required to reduce the HIV-1 RT-associated RNase H activity by 50%. ^b^ Compound concentration required to inhibit the HIV-1 IN catalytic activity by 50% in the presence of LEDGF. ^c^ Percentage of control activity measured in the presence of the indicated drug concentration.

**Table 4:** Quantitative analysis of compounds in pomegranate extracts characterized by inhibitory activities towards RT-associated HIV-1 RNase H and HIV-1 integrase LEDGF-dependent strand transfer.

Compounds	PGL		PGB		PGP	
	mg/g ^1^	RSD% ^2^	mg/g	RSD%	mg/g	RSD%
Ellagic acid	31.06	0.37	22.65	2.67	16.81	0.21
Luteolin	Tr ^3^	/	/	/	/	/
Apigenin	Tr ^3^	/	/	/	/	/
Punicalin α + β	/	/	15.80	0.78	2.51	0.24
Punicalagin α + β	/	/	76.06	0.75	29.51	0.25

^1^ mg of quantified compound/g of extract; ^2^ RDS%, relative standard deviation; ^3^ Tr, traces < 0.05 mg/g.

## Data Availability

All data are presented in this report.

## Supplementary Materials

The following are available online at https://www.mdpi.com/article/10.3390/plants10102124/s1, Figure S1: Representative LC-PDA profiles of PGL (A), PGB (B), and PGP (C) extracts (λ = 370 nm), Table S1: Selected method for quantification and specifications, linearity range, R2, and calibration curve of the main compounds quantified in pomegranate extracts through HPLC-PDA-MS/MS.
