# Supplementary material for: In Vitro Anti-HIV-1 Reverse Transcriptase and Integrase Properties of Punica granatum L. Leaves, Bark, and Peel Extracts and Their Main Compounds
Source: Plants (Basel). 2021 Oct 7;10(10):2124. doi: 10.3390/plants10102124 (PMC8539310; doi:10.3390/plants10102124)

Table S1. Selected method for quantification and specifications, linearity range, R^2 and calibration curve of the main compounds quantified in pomegranate extracts through HPLC-PDA-MS/MS.

Compound	Method	Linearity range (μg/mL)	R^2	Calibration curve
**Ellagic acid**	UV<br>370 nm	0.1-100	0.999	y = 44443.1x – 8,761.63
**Luteolin**	SRM$^+$<br>287→153	0.1-5	0.999	y = 1181554.7x + 61020.8
**Apigenin**	SRM$^+$<br>271→153	0.1-5	0.999	y = 1072063.8x + 545489.2
**Punicalin α+β**	UV<br>370 nm	5-100	0.999	y = 5759.8x – 9501.1
**Punicalagin α**	UV<br>370 nm	10-500	0.999	y = 1834.8x – 11147
**Punicalagin β**	UV<br>370 nm	10-500	0.998	y = 2425.9x – 1031

Figure S1. Representative LC-PDA profiles of PGL (A), PGB (B) and PGP (C) extracts (λ=370nm)

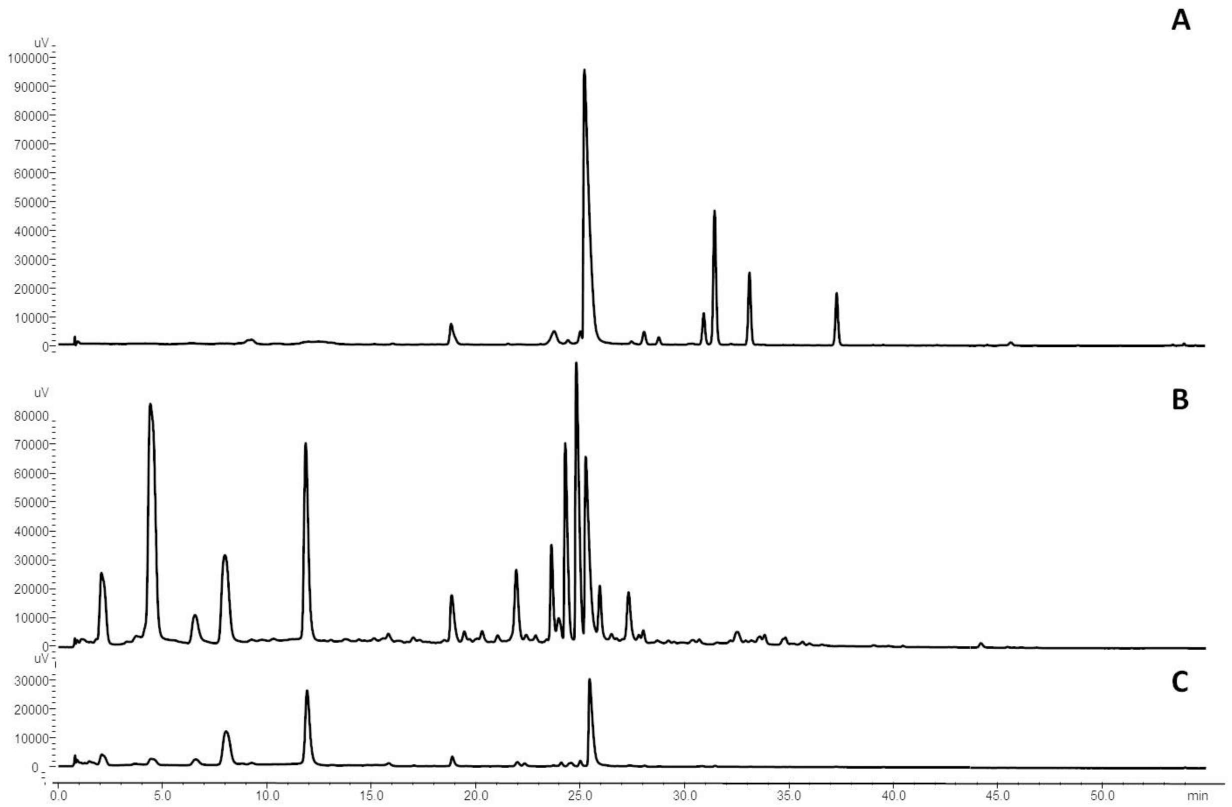

Supplement: Supplementary file 1 [file plants-10-02124-s001.zip › Supplementary materials.pdf]
