# Supplementary figures and images for: In Vitro Anti-HIV-1 Reverse Transcriptase and Integrase Properties of Punica granatum L. Leaves, Bark, and Peel Extracts and Their Main Compounds
Source: Plants (Basel). 2021 Oct 7;10(10):2124. doi: 10.3390/plants10102124 (PMC8539310; doi:10.3390/plants10102124)

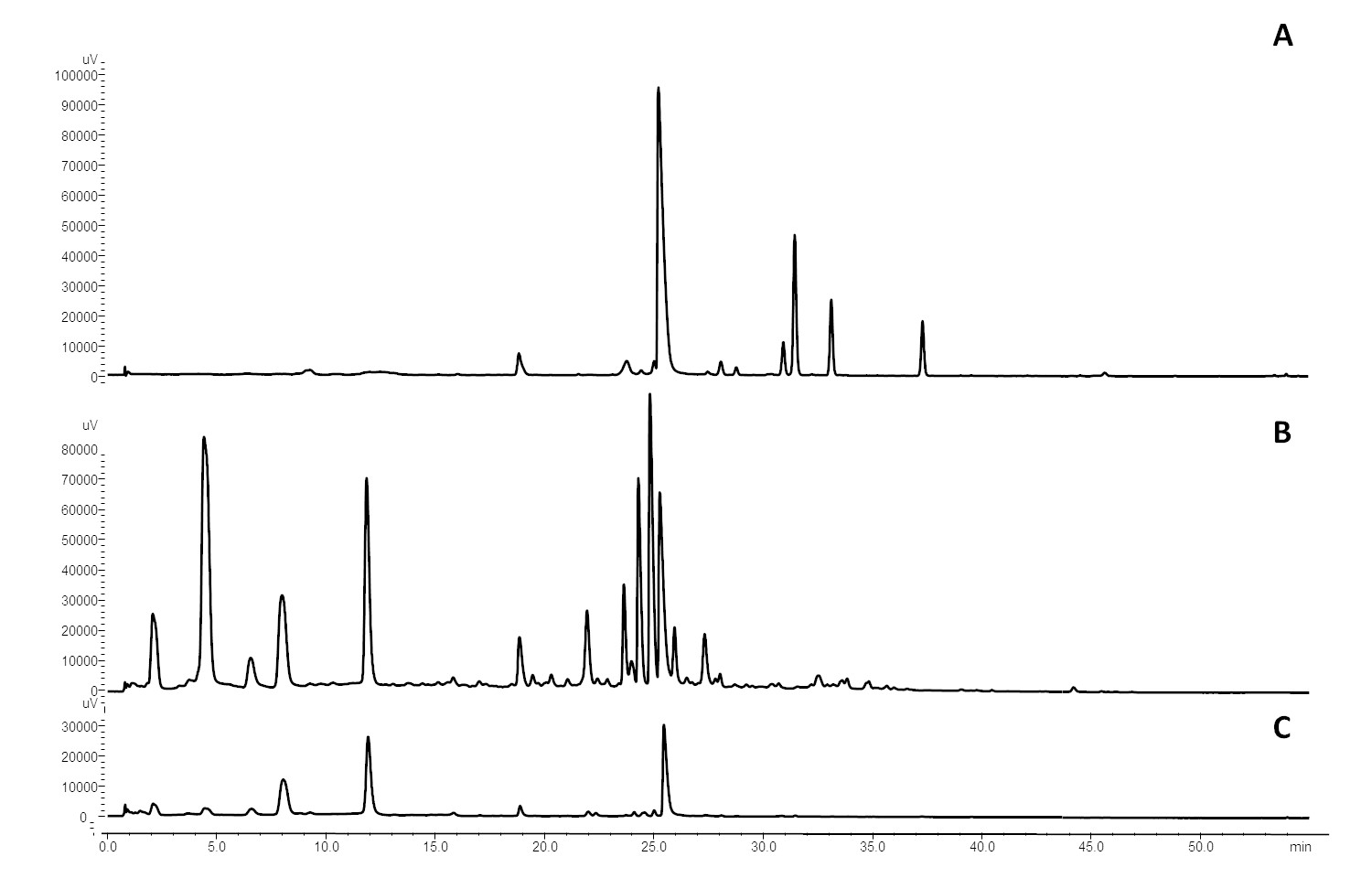

Supplement: Supplementary file 1 [file plants-10-02124-s001.zip › FigureS1.jpg]
